# Insights into the Affordable Medicines Facility-malaria in Ghana: the role of caregivers and licensed chemical sellers in four regions

**DOI:** 10.1186/s12936-016-1307-5

**Published:** 2016-05-10

**Authors:** Andrew A. Adjei, Peter Winch, Amos Laar, David J. Sullivan, Kwame S. Sakyi, Judith K. Stephens, George O. Adjei, Isaac A. Boateng, Vivian N. Ama Aubyn, Chrysantus Kubio, Julliette Tuakli, Linda Vanotoo, Bernard B. Bortei, Maame Amo-Addae, Felix Sorvor, Nathaniel Coleman, Sarah Dalglish, Richmond Owusu, Tsega Gebreyesus, Edward Essuman, Rebecca Greene, Ezekiel Ankomah, Kiely Houston, Constance Bart-Plange, Samuel Salamat, Ebenezer A. Addison, Isabella A. Quakyi

**Affiliations:** Office of Research, Innovation and Development, University of Ghana, Legon, Accra, Ghana; Department of International Health, Social and Behavioural Interventions Program, Johns Hopkins Bloomberg School of Public Health, 615 N. Wolfe St, Baltimore, MD 21205 USA; Department of Population, Family, and Reproductive Health, School of Public Health, University of Ghana, Legon, Accra, Ghana; Department of Molecular Microbiology and Immunology, Johns Hopkins Bloomberg School of Public Health, 615 N. Wolfe St, Baltimore, MD 21205 USA; Department of Biological, Environmental and Occupational Health Sciences, School of Public Health, College of Health Sciences, University of Ghana, P.O. Box LG 13, Legon, Accra, Ghana; Centre for Tropical, Clinical, Pharmacology and Therapeutics, School of Medicine and Dentistry, College of Health Sciences, University of Ghana, Legon, Accra, Ghana; Ashanti Akim Central Municipal Health Directorate, Ghana Health Services, Konongo, Ghana; National Malaria Control Programme, Ministry of Health, Accra, Ghana; Gonja Health Directorate, Ghana Health Services, Accra, Ghana; Child and Associates, Accra, Ghana; Regional Health Directorate, Ghana Health Services, Accra, Ghana; Shama District Health Directorate, Ghana Health Services, Shama, Ghana; Department of Paediatrics, Johns Hopkins University School of Medicine, Baltimore, MD USA; Kpone Katamanso District Health Directorate, Ghana Health Services, Kpone, Tema, Ghana

**Keywords:** Affordable Medicines Facility-malaria, Caregivers, Licensed chemical sellers, Artemisinin-based combination therapy

## Abstract

**Background:**

The Affordable Medicine Facility-malaria (AMFm) was an innovative global financing mechanism for the provision of quality-assured artemisinin-based combination therapy (ACT) across both the private and public health sectors in eight countries in sub-Saharan Africa. This study evaluated the effectiveness of AMFm subsidies in increasing access to ACT in Ghana and documented malaria management practices at the household and community levels during the implementation of the AMFm.

**Methods:**

This study, conducted in four regions in Ghana between January, 2011 to December, 2012, employed cross-sectional mixed-methods design that included qualitative and quantitative elements, specifically household surveys, focus group discussions (FGD) and in-depth interviews.

**Results:**

The study indicated high ACT availability, adequate provider knowledge and reasonably low quality-assured ACT use in the study areas, all of which are a reflection of a high market share of ACT in these hard-to-reach areas of the country. Adequate recognition of childhood malaria symptoms by licensed chemical seller (LCS) attendants was observed. A preference by caregivers for LCS over health facilities for seeking treatment solutions to childhood malaria was found.

**Conclusions:**

Artemisinin-based combination therapy with the AMFm logo was accessible and affordable for most people seeking treatment from health facilities and LCS shops in rural areas. Caregivers and LCS were seen to play key roles in the health of the community especially with children under 5 years of age.

## Background

The Affordable Medicine Facility-malaria (AMFm) was an innovative global financing mechanism for the provision of quality assured artemisinin-based combination therapy (ACT) across both the private and public health sectors in eight countries in sub-Saharan Africa. The mechanism provided a subsidy at the manufacturer level together with a set of supporting interventions. A green leaf logo is affixed to all ACT drugs supported by the AMFm programme for easy identification and quality assurance. The objectives of the AMFm were to expand access to quality-assured ACT (QAACT) and to delay resistance to artemisinin monotherapies [1].

Ghana was one of the eight implementing countries of the AMFm initiative [2]. The others were Kenya, Cambodia, Madagascar, Niger, Nigeria, Tanzania and Uganda. Implementation began in August, 2010 when the first batch of QAACT was brought into the country. A total of 52.7 million treatment doses of the subsidized ACT was imported into Ghana from August, 2010 to December, 2012. Majority (48.1 million) was brought in by the private sector representing 90 % of total orders [3]. The private-for-profit health care sector in Ghana includes licensed chemical sellers (LCS) who are also the first point of care for febrile illnesses, especially in remote and hard-to- reach areas. There are more than 10,000 LCS shops in the Ghana [4].

As in most countries in the sub-region, the general population of Ghana is at risk for malaria but the most vulnerable include children under 5 years old and pregnant women [5], hence the role of caregivers in providing prompt and effective care for children under-five is critical. This role is endorsed by the National Malaria Control Programme (NMCP), which is the main government agency responsible for prevention and control of malaria in Ghana. The main prevention and control measures used in sub-Saharan Africa are: promotion of use of insecticide-treated nets (ITNs); indoor residual spraying (IRS); intermittent presumptive treatment for pregnant women with sulfadoxine-pyrimethamine (SP) and early detection and prompt treatment of malaria [5–8]. There is considerable evidence of utilization of these interventions [9] whose implementation has led to improvement in related health indicators [10]).

The Global Fund conducted an independent evaluation of the AMFm pilot in all eight countries from 2009 to 2011 and showed that the concept was successful [11]. However, countries continued to implement the AMFm until December, 2012. This study, therefore, sought to evaluate the AMFm programme in Ghana between 2011 and 2012. The main objectives of the study were to: (i) evaluate the effectiveness of AMFm subsidies in increasing access to ACT in Ghana and, (ii) document malaria management practices at the household and community levels during the implementation of the AMFm. As an extension to previous work done [12, 13], this study also focuses on the role of key stakeholders such as caregivers, health care providers as well as LCS in the hard-to-reach communities.

## Methods

### Study design

This study employed cross-sectional mixed methods that included qualitative and quantitative elements. Household surveys, focus group discussions (FGD) and in-depth interviews were carried out between January, 2011 to December, 2012.

### Study setting

This study was conducted in five sites from four of the ten regions of Ghana: they are the Asante-Akim North and South Districts in the Ashanti Region, Shama District in the Western Region, Kpone-Katamanso District in the Greater Accra Region, and West Gonja District in the Northern Region. These study sites are located in the three ecological zones (Forest, Coastal, and Savannah) in Ghana. Asante-Akim North and South Districts are located in the Forest Zone and occupy 1462 km^2^ and 1217.7 km^2^ of land, respectively. The Shama district occupies an area of 384 km^2^ and it is located within the Coastal-Forest zone while the Kpone-Katamanso District, which occupies an area of 215.4 km^2^, is located in the Coastal-Savannah zone. The West Gonja District occupies an area of 17,317 km^2^ and is located in the Savannah zone. The main economic activities of the selected districts are subsistence farming, animal husbandry and fishing. However, a considerable number of the inhabitants in all the study sites engage in petty trading. Aside from the differences in ecology the population in the study sites have different socio- cultural backgrounds.

### Data collection

#### Study participants

These included caregivers of children under-five, LCS and health care providers. A total of 285 caregivers were enrolled (Asante-Akim North, N = 57; Asante-Akim South, N = 54; Shama, N = 60; Kpone-Katamanso, N = 54; and West Gonja, N = 60). All LCS in the study sites were enrolled, N = 149 (Asante-Akim North, N = 36; Asante-Akim South, N = 34; Shama, N = 36; Kpone-Katamanso, N = 34; West Gonja, N = 9). Twenty-one health care providers also participated.

#### Study procedure

Appropriate sensitization was done through community durbars and meetings to explain the purpose of the study and answer questions at the suitable literacy level. Informed consent was obtained from each study participant.

Data collection for the study comprised household surveys, FGD and in-depth interviews. Structured questionnaire were administered to ascertain respondents’ views on relevant themes. For caregivers, the questionnaire sought to ascertain knowledge on malaria, first point of seeking care for malaria cases in children, attitude towards malaria prevention, control and treatment. The questionnaire for LCS sought to determine their role in promoting treatment compliance to ACT, correct drug administration, and their perceptions on efficacy and availability of ACT in the treatment of malaria in children under 5 years of age. Health care providers were interviewed using an interview guide to find out their views on the circumstances for prescribing ACT and other anti-malarial and in addition their prescribing behaviour was observed. The guide also sought information on the perception of health care providers on the availability, quality and reach of the AMFm ACT. The health care providers were community health nurses, disease control officers, physicians and pharmacists.

Twenty FGDs were held across the five districts. Each FGD comprised 8–12 individuals per session. For each site there were four FGDs; two male and two female groups of caregivers. The caregivers included mothers, fathers, grandparents, and other extended family members. The male and female groups were separated to allow freedom of expression as socio-cultural practices limits females’ expression and participation in the presence of their male counterparts. The female caregivers were randomly selected at the outpatient departments (OPD) of health facilities and women’s groups in the communities. On the other hand, male participants were recruited at the household level; this is because they are not the ones who usually send their under-5 year old children to the health facility for treatment.

Semi-structured and in-depth interviews were conducted for the LCS to determine their role in the AMFm activities in Ghana, including awareness of AMFm, preference or otherwise for specific anti-malaria medicines including AMFm ACT, ease of access and cost of ACT, perceptions on efficacy and quality, and perceptions on preference of community members for anti-malaria medication.

The views of policy makers were sought on the AMFm supply chain management, and the quality and availability of health care at the rural levels in Ghana. These officials were purposively sampled and were given self-administered questionnaire. In-depth interviews were held with five health policy officials at the national level on their role and contribution to malaria diagnosis and treatment.

### Ethical consideration

Ethical approval for the study was obtained from the Ghana Health Service Ethical Review Committee (GHS-ERC:10/7/11), the University of Ghana School of Medicine and Dentistry Ethical and Protocol Review Committee (MS-Et/M.11-P.5.3/2010-11) and the Johns Hopkins Bloomberg School of Public Health Institutional Review Board, Maryland, USA (IRB 3482). Written informed consent was obtained from all participants. During the process, prospective participants were assured that the information they provide will be treated with strict confidentiality.

### Statistical analysis

Data were doubly entered and afterwards validated to ensure consistency. Univariate analysis produced descriptive statistics presented as proportions. Bivariate analysis was done to determine associations between the outcome variable (access to AMFm ACT measured by affordability, availability and market share) and selected explanatory variables. All analyses were performed using SPSS Version 15.0 (SPSS Inc. Chicago). *P* value less than 0.05 was considered statistically significant.

## Results

Table 1 presents the characteristics of the different participants (caregivers, LCS attendants, and healthcare providers) of the study. The majority (77.1 %) of the caregivers were mothers of children under 5 years old. Of the 105 caregivers, 101 responded to the question on literacy out of these 54 % could read and write in English. About 70 % of the caregivers were employed in the informal sector and they included hairdressers, fishmongers, seamstress, traders and farmers. LCS attendants were mostly males, and tended to be married or cohabiting. Over 90 % of the LCS attendants had completed at least Senior High School education. In addition, most (81 %) had received training on malaria symptom recognition and treatment protocols from the Pharmacy Council and the NMCP.

**Table 1:** Characteristics of study participants by type

	Asante Akim North N (%)	Asante Akim South N (%)	Shama N (%)	Kpone Katamanso N (%)	Gonja West N (%)	Total N (%)
Type of respondent/participant						
Caregivers						
Type of caregiver	N = 20	N = 19	N = 20	N = 21	N = 25	N = 105
Mother of young child	11 (55)	16 (84.2)	19 (95)	14 (66.7)	21 (84)	81 (77.1)
Father of young child	5 (25)	2 (10.5)	0	4 (19)	4 (16)	15 (14.3)
Other	4 (20)	1 (5.3)	1 (5)	3 (14.3)	0	9 (8.6)
Literacy of caregivers	N = 20	N = 18	N = 19	N = 20	N = 24	N = 101
Cannot read and write English	4 (20)	10 (55.6)	13 (68.4)	5 (25)	15 (62.5)	47 (46.6)
Can read and write English, but with difficulty	6 (30)	5 (27.8)	2 (10.6)	9 (45)	5 (20.8)	27 (26.7)
Can read and write English easily	10 (50)	3 (16.7)	4 (21)	6 (30)	4 (16.7)	27 (26.7)
Occupation of Caregivers	N = 20	N = 19	N = 20	N = 21	N = 25	N = 105
Unemployed	1 (5)	1 (5.30	1 (5)	0	0	3 (2.9)
Formal sector	5 (25)	4 (21)	0	2 (9.5)	3 (12.0)	14 (13.3)
Informal sector	14 (70)	14 (74)	19 (95)	19 (90.5)	22 (88.0)	88 (83.8)
LCS attendants						
Gender	N = 19	N = 20	N = 20	N = 20	N = 10	N = 89
Male	15 (78.9)	16 (80.0)	13 (65.0)	12 (60.0)	9 (90.0)	65 (73.1)
Female	4 (21.1)	4 (20.0)	7 (35.0)	8 (40.0)	1 (10.0)	24 (26.9)
Marital status	N = 19	N = 20	N = 20	N = 20	N = 10	N = 89
Married/cohabiting	9 (47.4)	14 (70)	14 (70.0)	8 (40.0)	7 (70.0)	52 (58.4)
Divorced/separated	2 (10.5)	4 (20)	1 (5.0)	0	0	7 (7.9)
Never married/never co-habited	8 (42.1)	2 (10)	5 (25.0)	12(60.0)	3 (30.0)	30 (33.7)
Education	N = 19	N = 20	N = 20	N = 20	N = 10	N = 89
Primary education	4 (21.1)	1 (5.0)	1 (5.0)	1 (5.0)	1 (10.0)	8 (9.0)
Secondary/SHS	11 (57.8)	13 (65.0)	12 (60.0)	11 (55.0)	8 (80.0)	55 (61.8)
Tertiary	4 (21.1)	6 (30.0)	7 (35.0)	8 (40.0)	1 (10.0)	26 (29.2)
Receipt of LCS training	N = 19	N = 20	N = 20	N = 20	N = 10	N = 89
Yes	8 (42.1)	20 (100)	20 (100)	16 (80)	10 (100)	74 (83.1)
No	11 (57.9)	0	0	4 (20)	0	15 (16.9)
Health care providers						
Nurse prescriber	–					11 (52.4)
Physician assistant						7 (33.3)
Pharmacy technician						2 (9.5)
Laboratory technologist						1 (4.8)
Gender						
Male	–					7 (33.3)
Female						14 (66.7)
Length of practice						
Up to 5 years	–					10 (47.6)
6–10 years						2 (9.5)
Above 10 years						9 (42.9)

**Fig. 1:**
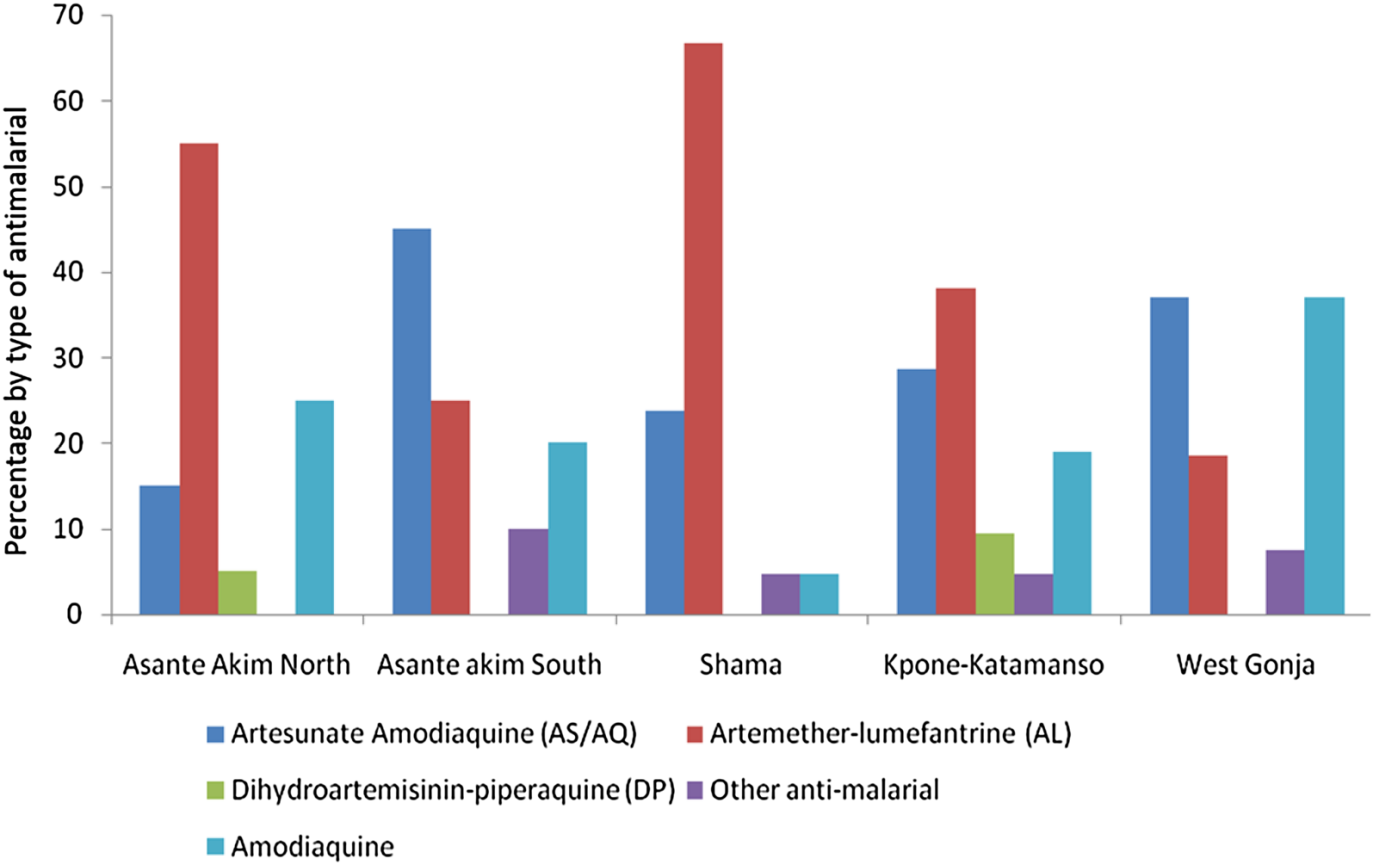
Distribution of anti-malarial drugs clients purchased at LCS by study site. The *figure* presents the distribution of anti-malarial drugs that caregivers of children under 5 years had purchased from LCS by study site. Exit interviews were conducted with caregivers, who had purchased anti-malarial drugs for their children under 5 years. The range of drugs included AS/AL, AL, DP, and Amodiaquine as detailed in the legend below

At the time of this survey, ACT was the most frequently purchased anti-malarial at the LCS. The most popular ACT at the time was artemether-lumefantrine. Of note, however, were the purchase of monotherapies (Amodiaquine) and some other non-ACT anti-malarial. The frequency of anti-malarial medicines by study site is depicted in Fig. 1.

Across the four regions there was a trend towards fewer LCSs located within 100 metres of a health facility. Overall, there were fewer LCSs that had in stock the ACT with the AMFm Green Leaf logo. However, among those who stocked the AMFm ACT, there were more LCSs selling at 1.50 Ghana cedis or less, which was the recommended selling price. Thus, ACT was generally affordable, but not necessarily accessible. This did not significantly differ by study site (P > 0.05; Table 2).

**Table 2:** Access to, and affordability of AMFm ACTs as measured by proximity, price, and availability in the study sites

Characteristics	Districts					Total	Chi square	P value
	Asante-Akim North	Asante-Akim South	Shama	Kpone Katamanso District	West Gonja			
Total number of LCS per district	N = 36 (%)	N = 34 (%)	N = 36 (%)	N = 34 (%)	N = 9 (%)	149		
LCS distance to the health facility (proximity)								
Up to 100 m	8 (22.2)	11 (32.4)	2 (5.6)	8 (23.5)	3 (33.3)	32 (21.5)	8.643	0.071
More than 100 m	28 (77.8)	23 (67.6)	34 (94.4)	26 (76.5)	6 (66.7)	117 (78.5)		
Pricing of ACTs	N = 20	N = 20	^†^	N = 20	N = 27			
Affordable (GHC 1.5 or less)^a^	16 (80.0)	11 (55.0)	^†^	14 (70.0)	17 (63.0)	58 (66.7)	3.092	0.378
Not affordable (more than GHC 1.5)	4 (20.0)	9 (35.0)	^†^	6 (30.0)	10 (37.0)	29 (33.3)		
Availability of AMFm ACTs	N = 20 (%)	N = 21 (%)	N = 22 (%)	N = 20 (%)	N = 8 (%)	91		
ACTs with green leaf logo	5 (25.0)	5 (23.8)	6 (27.3)	4 (20.0)	0 (0.0)	20 (22.0)	2.806	0.591
Others	15 (75.0)	16 (76.2)	16 (72.7)	16 (80.0)	8 (100)	71 (78.0)		

^†^There were no data recorded on Affordability for Shama district

Table 3 summarizes the malaria management practices of caregivers of children under 5 years. Caregivers purchase of anti-malarial for children under 5 years were motivated by various signs including hot body (38 %), vomiting (14 %), and loss of appetite (17 %). About 2 % of the caregivers sought anti-malarial from LCSs for their convulsing children. The drugs bought were mostly tablets (44 %), syrups (26 %), and dispersible forms (25 %). Generally, caregivers intended to administer drugs bought orally.

**Table 3:** Malaria management practices of caregivers, N = 285

Description	N (%)
Specific symptom that informed purchase of a drug	
Hot body	102 (37.8)
Yellow urine	18 (6.7)
Vomiting	39 (14.4)
Body pains	10 (3.7)
Diarrhoea	9 (3.3)
Headache	32 (11.9)
Convulsion	4 (1.5)
Loss of appetite	45 (16.7)
Other	11 (4.1)
Main reason you bought this particular drug	
Shopkeeper recommended	73 (67.6)
Friend/neighbour/family	5 (4.6)
It works better than other drugs	20 (18.5)
Cost	3 (2.8)
Was recommended by a health worker	2 (1.9)
Other	5 (4.6)
Formulation of drug purchased	
Tablet	47 (43.5)
Syrup	28 (25.9)
Dispersible	27 (25.0)
Other	6 (5.6)
How will you give (or take) the medicine	
Oral (tablet)	34 (37.8)
Oral (dispersible)	43 (47.8)
Injection	1 (1.1)
Others	12 (13.3)

### Malaria management practices by caregivers, LCS and health care providers

#### Licensed chemical sellers

The LCS engaged the customers in an interaction by asking questions about whom the drug was being bought for. Various questions were asked including, the body temperature of the child, age of the person going to take the drug and signs and symptoms the person is presenting and whether the patient has diarrhoea.

Below are some of the questions as reported by their clients:

- *“He asked me of the signs of the illness and the number of days the child had been sick?”*
- *“He asked me whether I had given the child any drug before coming?”*
- *“Is the child able to breastfeed? Does she vomit?”*
- *“The shopkeeper asked me if the child had really lost appetite, very hot and was not playing?”*

After selling the drug to the customers most of the LCS gave information about the drug purchased. Some of the information given include;

- *“Give it two times in a day for three days. Give 10mls at a time.”*
- *“Give morning and evening. Give after meals with plenty water.”*
- *“Give the child food before administering the drug. Give half white and half yellow at a time for two times in a day for three days.”*
- *“Give two times in a day for two days and come for the third drug some other time. Give half yellow, half white at a time.”*
- *“He said when the child takes the drug and feels weak I should not worry is the nature of the drug and I should not give more than the right dosage.”*
- *“Should be dissolved in water for twice daily before giving to the child.”*
- *“The drug should be kept in dry place and remain in the box (package). The tablet should be dissolved in small amount of water before giving to the child.”*
- *“The shop keeper told me that, this drug is effective in treating malaria and is less expensive and has the green leaf logo on it.”*

#### Caregivers

Caregivers in this study prefer seeking care at LCS shops as against the health facilities. Caregivers indicated reasons for their choices bothering on the timing of the illness, proximity to LCS and unbearable duration of time spent at the health centre.

- *“I don’t have time to go and queue at the hospital.”*
- *“Because it was closer to my house, compared to the health centre.”*
- *“It was in the night, when I send him to hospital I don’t think I will meet somebody there, so I took him to the drug store.”*
- *“It was a Saturday and I didn’t know whether the hospital was working or not and I didn’t want to keep her until Monday so I decided to send her to the drug store.”*
- *“The child’s body was hot and the drug shop is close to my house so I decided to go and complain to the LCS.”*

#### Cost and affordability of AMFm drugs

Caregivers were asked why they decided to go the drug shop and various responses were elicited. Some of the reasons given by the caregivers that related to finances and health insurance were;

- *“I didn’t have enough money at home as at the time the symptoms started so I decided to get first aid at the drug shop”.*
- *“Because I don’t have Health Insurance”.*
- *“Child’s NHIS card was in father’s room which was locked.”*
- *“I do not have insurance so I went to the drug shop so that the cost will be lesser than the hospital”.*
- *“I do not have money to take my child to the hospital so I decided to buy drugs from the drug store.”*

Other reasons given by the caregivers which related to experience with that particular drug, for first aid and trust for the LCS prescription amongst others were;

- *“After few days the symptoms didn’t stop but had worsened”.*
- *“Because after given the paracetamol syrup the temperature did not go away”.*
- *“Because I normally go there before the need for hospital.”*
- *“Best place to visit for immediate support or medical assistance.”*
- *“Even the hospital approves of that shop and the price of the hospital and the shop is the same.”*
- *“I decided to go to the drug shop because my child was crying each and every day and was not eating any food I gave him.”*
- *“I decided to go to the drug store because the first time my child developed that same conditions, a friend of mine (a nurse) introduced me to the drug and it worked very well for me.”*
- *“I think going to the drug shop is important because the same drugs would have been given if I had gone to the hospital or health centre.”*
- *“I was using it as first aid so that if the symptoms do not go I will visit the hospital.”*
- *“I went there because I have seen them to be qualified people.”*
- *“I was given the same treatment when I went to the hospital that is why I decided to go to the shop the second time.”*
- *“That woman is very good and she gives good treatment.”*

Caregivers were thereafter questioned as to why or not they thought they were given a good treatment for their children’s illness. Some of the responses given were;

- *“After giving the drug I noticed the hotness and cold went away.”*
- *“After the first dose, the child’s headache stopped.”*
- *“Because it stopped the fever of the child.”*
- *“Because my child’s rise in temperature went down, her urine came back to its normal colour”.*
- *“Because the child has started playing and eating.”*
- *“Because the fever and vomiting went away.”*
- *“The child became well after the full course.”*
- *“The fever subsided after the first dose.”*

When asked about how they knew their children were well again, caregivers attributed various reasons. Most of the caregivers said they knew because their children started playing again, child’s temperature reduced, child was able to breastfeed and because their children stopped vomiting and crying. Most of the caregivers, when asked whether they will return to the drug shop, responded that they will return.

### National level policy makers’ views on commodity supply chain management, availability and quality

The views of national level policy makers on the above subject, and their contribution to ensuring a smooth supply of quality-assured malaria prevention and treatment commodities are summarized below.

### AMFm supply chain management

Policy makers at the national level are seen to contribute greatly to AMFm supply chain management in malaria control. This involves program design, implementation, supervision and coordination of procurement processes. Policy makers regulate activities of both the public and private healthcare levels in Ghana. Some of the responses given by policy makers in relation to their involvement in AMFm supply chain management are as follows;

*“My roles include providing technical****s****upport in developing policies and guidance for malaria control. Help in resource mobilization, monitoring and supervision” (Policy Maker 1).*

*“We ensure that ACTs are in country, are available at all facilities, and are sold at the right prices by negotiating with all stakeholders. We also explore the right public and private channel for distribution of drugs” (Policy Maker 2).*

*“It is our role to ensure that effective preventive interventions and anti*-*malaria medicines are available and accessible to the entire population” (Policy Maker 3).*

“There is a task team for c*oordinating all activities of the AMFm, marketing, training of health officials, ensuring drug safety and quality and liaising with all stakeholders to ensure effective functioning of the program” (Policy Maker 4).*

### Improving quality and availability of care in Ghana

Policy makers were of the view that quality and availability of care involves adhering to the right policies and guidance principles of the health service. Availability and proper coordination of resources contributes efficiently to the quality of care and this involves training of healthcare practitioners in ensuring proper diagnosis before treatment, sourcing or procuring malaria medications directly from WHO or WHO-accredited outlets were also considered relevant by policy makers.

*“It is my view that there are good policies, protocols, and guidelines already in place for quality of health care in Ghana and all that is needed is ensuring compliance” (Policy Maker 1).*

*“In improving quality of care, there should be better commitment and effort from all those involved in malaria management” (Policy Maker 2).*

*“To ensure quality of drugs for health care, procurements are only made from World Health Organization’s (WHO) approved sources” (Policy Maker 3).*

*“In improving quality of care and treatment of malaria, there should be the availability and use of Rapid Diagnostic Test (RDT) in remote areas for testing of all cases before treatment, I think it is effective. It is better than presumptive diagnosis, its time saving and easy to use” (Policy Maker 4).*

*“Ensure households get prompt access to malaria treatment, home based care, partnering with agencies and training of CHW. Ensuring quality ACTs available at affordable prices and link diagnosis to treatment of malaria” (Policy Maker 5).*

## Discussion

As an innovative global financing mechanism for the provision of QAACTs across the private as well as the public health sectors in high malaria endemic settings, the AMFm programme sought to expand access to potent malaria management medications and ultimately improve case management and possibly influence the rate of resistance development to currently recommended anti-malarial. This study sought to evaluate the potential impact that AMFm subsidy and related policies have had on QAACT accessibility and childhood malaria management practices in hard-to-reach areas of Ghana where health infrastructure and systems are weak. The study evaluated the programme in Ghana, one of the eight AMFm implementing countries and was done after the official end of the programme to assess potential beneficial impact beyond the policy implementation period. This study also provides a unique insight on the anti-malarial market and extended the possible policy ramifications of the AMFm strategy by including viewpoints from key stakeholders such as health policy managers and national disease control officers.

The findings from the study indicate high ACT availability, adequate provider knowledge and reasonably low QAACT process in the study areas, all of which are a reflection of a high market share of ACT in these hard-to-reach areas of the country. The findings indicating high artesunate -amodiaquine (AA) and artemether-lumefantrine (AL) availability reflect the relative longer existence of these respective ACT in Ghana to the 2005 ACT policy implementation which recommended AA as first-line treatment for uncomplicated malaria and AL as an alternative for those who do not tolerate AA (Malaria Control Programme Policy 2005). The relatively low dihydroartemisinin-piperaquine availability reflects the fact that this ACT though among the recommended first-line treatments for uncomplicated malaria in Ghana, was only introduced later (in 2009) when the original policy was reviewed.

These findings of widespread AMFm QAACTS in hard-to-reach areas in Ghana, therefore, do not confirm expressed fears in certain quarters regarding the possibility of market profiteering by middlemen in the ACT distribution chain. The findings also partially negate the notion of market forces in the private sector being necessarily problematic in relation to ACT availability. However, the somewhat enduring presence of monotherapies, particularly that of amodiaquine is worrying and may be due to the particular emphasis that health education messages aimed at discouraging anti-malarial monotherapies laid on artemisinin monotherapies *per se*. This messaging strategy, while laudable and an important effort to protect these crucial anti-malarial may have inadvertently deflected the focus from other important (availability-wise) anti-malarial, such as amodiaquine, which has been in use in Ghana for a long time. These considerations are supported by the finding from previous studies indicating artesunate monotherapy as the second topmost selling anti-malarial in urban Nigeria [14]. The continuous presence of anti-malarial monotherapies in hard-to-reach-areas of the country, in spite of the fact that regulations discouraging manufacture, distribution, importation or sale of anti-malarial monotherapy are in operation in Ghana is a rallying call for strengthening applicable regulatory implementation procedures to the supply chain management and stricter enforcement of the applicable market authorization regulations regarding these anti-malarials.

The data indicating adequate recognition of childhood malaria symptoms by LCS attendants also points towards appreciable familiarity with other relevant global childhood initiatives such as the integrated management of new-born and childhood illness (IMNCI) standards and requirements. While the encouraging findings indicating that majority of LCS attendants provided specific and adequate pre-dosing preparation instruction, or appropriate dosing and storage information to caregivers is likely due to satisfactory internalization of the widely-circulated instructional and promotional messages that accompanied the AMFm green leaf logo implementation programme, the findings indicating that some LCS attendants in some of the study areas did not sell ACT to caregivers, or sold either incomplete courses of anti-malarial medications or gave instructions inconsistent with complete dosing is worrisome. These LCS attendants were in the minority but the potential implications of selected expressions (e.g., “give two times in a day for two days and come for the third dose some other time”) on the possibility of perpetuating the resistance-promoting sub-therapeutic anti-malarial drug concentrations should not be disregarded. Thus, education and training activities aimed at improving malaria management practices should be appropriately targeted and intensified. This also suggests that a more rigorous implementation of malaria management practice with specific information on the dangers and implications of under-dosing or incomplete treatment of malaria should be undertaken at the national level, preferably through appropriate additions to the Standard Treatment Guidelines.

The relatively lower AMFm ACT prices confirm expectations and findings from previous studies concluding that AMFm medications are less expensive than their counterparts [14], and is a reflection of the high market penetration of QAACT even in these hard-to-reach areas and thus of the impact of the AMFm policy. However, since the AMFm goal of increasing access has not sufficiently extricated the concept of availability from affordability, both of which are viewed as critical to accessibility, the close overlap makes distinguishing between some of the associated variables problematic. While unaffordability is a major barrier to accessing ACT in countries where malaria is widespread [15, 16], the findings from this study that the national level price recommended for retailing AMFm QAACT at [1.50 Ghana cedis ($ 0.40) or less] was considered by majority of the participants as affordable. Thus, even in these hard-to-reach areas where the majority of the population could be described to be of low socio-economic status, QAACT provided through the AMFm programme were affordable [17], confirming the impact of the policy. However, not all retailers (with few exceptions) were selling at the recommended price, thus, raising affordability concerns for those without health insurance in these hard-to-reach areas. Overall, though, the findings generally confirm those from previous studies showing that AMFm ACT was less expensive than the non-AMFm counterparts [14].

From the point of view of some national health officials, the procurement process requirements of the AMFm programme requiring a global sourcing route resulted in regional health officials not able to obtain the entire QAACT requirements for the needs of their respective areas. This frequently raised the need to procure additional supplies of ACT from approved private sector suppliers. However, this raised availability as well as affordability concerns as it resulted at times in increased cost of the AMFm drugs.

The study findings indicating a preference by caregivers for LCS over health facilities for seeking treatment solutions to childhood malaria is consistent with findings from previous studies from Ghana [18, 19]. These findings also corroborate findings from earlier studies from Ghana indicating that majority of treatments for childhood malaria occurs outside the public health system [20], and with findings from elsewhere concluding among others that, mothers are capable of treating childhood malaria at home [21]. The viewpoints expressed in one of the FGD to the effect that *I was given the same treatment when I went to the hospital that is why I decided to go to the shop the second time*, is particularly interesting and merits further studies to explore the underlying reasoning, as well as potential implications of such assertions. The importance of the other sentiments relating to affordability and cost of health care including those indicating a higher preference for the LCS rather than the health facility for childhood malaria symptoms, together with the FGD statement(s) to the effect that *I did not have enough money at home as at the time the symptoms started so I decided to get First Aid at the drug store*” or, “*I do not have insurance so I went to the drug shop so that the cost would be less than the hospital* further emphasize the critical need for financial barrier removal. In this respect, interventions aimed at enhancing registration with the National Health Insurance Scheme as well as improving effective implementation of the NHIS are all important determinants in ensuring easier access to childhood malaria management options.

The expressed viewpoints by some respondents of not having enough money hence their decision to visit the LCS rather than a health facility is consistent with findings from a previous study from Kenya [22], and with the general conclusions from other studies to the effect that the AMFm was indeed a good policy to increase access to QAACT for the poor [1, 23]. However, it is interesting to note that obvious logistic, systemic and perceived or real administrative barriers, including notions of overcrowding and long waiting hours at health centres were among some of the important factors that purportedly discouraged caregivers from seeking care at health facilities. While health facilities have closing hours, LCS are open at hours that are convenient to the community including weekends and holidays and may sometimes sell on credit basis to their customers [17]. These factors contribute to the provision of an obvious convenience advantage that is difficult to resist given the circumstances. Furthermore, the uneven distribution of health facilities in developing countries and the relative difficult health facility accessibility plays an influential role in the use of formal healthcare [24, 25]. This is confirmed by the findings from a study in Kenya indicating a reduction in the number of patients seeking care in formal health facilities as the distance from health facilities increased [24], and by the findings from studies in Burkina Faso and Tanzania showing that majority of the population obtains malaria medicines at licensed chemical shops [26–28], probably due to the same reason. The indirect cost of travelling long distance and long waiting time in health facilities are all factors that influence decision to visit LCS, therefore, underscoring the important role of removing physical and financial barriers to improve access to health care in countries such as Ghana.

Overall the findings from the study indicate the AMFm programme to be functional in Ghana and showing positive impact of the programme following its implementation in Ghana. Specifically, the AMFm policy contributed to widespread awareness and availability of ACT medications in hard-to-reach areas of the country. The findings also indicate that the AMFm policy most likely enhanced affordability of QAACT in rural areas of Ghana. The important role of primary caregivers and LCS in childhood malaria management was also validated.

It is worthy of note, however, that some of the findings presented be interpreted with caution. For instance, the actions of the sample of 89 LCS attendants from the five study districts of the four regions of Ghana may not be representative of all LCS in Ghana. Therefore, the LCS-related information provided may not be generalized beyond the study sites.

## Conclusions

Overall the AMFm programme was very functional and producing results in Ghana at the time of this study. ACT drugs were the drugs most commonly available, prescribed and sold in health facilities and in LCS shops. There was a widespread awareness of ACT drugs and the AMFm Green Leaf logo. It appears from these data that ACT was made affordable for most people seeking treatment from health facilities and LCS shops in rural areas. Caregivers and LCS were seen to play key roles in the health of the community especially with children under 5 years of age.

## Abbreviations

AMFm: Affordable Medicine Facility-malaria
ACT: artemisinin-based combination therapy
FGD: focus group discussions
LCS: licensed chemical sellers
QAACT: quality-assured act
ITNs: insecticide-treated nets
NMCP: National Malaria Control Programme
IRS: indoor residual spraying
SP: sulfadoxine-pyrimethamine
NHIS: National Health Insurance Scheme
IMNCI: integrated management of new-born and childhood illness

## References

[1] Arrow KJ, Panosian C, Gelband H (2004). Saving lives, buying time: economics of malaria drugs in an age of resistance.

[2] WHO. Q&A on the Affordable Medicines Facility malaria (AMFm). http://www.who.int/malaria/media/affordable_medicines_facility_qa/en/.

[3] National Malaria Control Programme [NMCP]. Briefing Notes to the Minister of Health on the Affordable Medicines Facility for Malaria (AMFM) in Ghana. 2012.

[4] Seiter A, Gyansa-Lutterodt M (2009). Policy note: the pharmaceutical sector in Ghana.

[5] National Malaria Control Programme [NMCP] (2015). Strategic Plan for Malaria Control in Ghana 2008–2015.

[6] President’s Malaria Initiative. Malaria Operational Plan Malawi FY. 2011.

[7] Gss G, Macro ICF (2009). Ghana demographic and health survey 2008.

[8] WHO (2010). World malaria report 2010.

[9] Owusu-Agyei S, Awini E, Anto F, Mensah-Afful T, Adjuik M, Hodgson A (2007). Assessing malaria control in the Kassena-Nankana district of northern Ghana through repeated surveys using the RBM tools. Malar J.

[10] Hommerich L, Von Oertzen C, Bedu-Addo G, Holmberg V, Acquah PA, Eggelte TA (2007). Decline of placental malaria in southern Ghana after the implementation of intermittent preventive treatment in pregnancy. Malar J.

[11] AMFm independent evaluation team. Independent Evaluation of Phase 1 of the Affordable Medicines Facility-malaria (AMFm), Multi-Country Independent Evaluation Report: Final Report. Calverton Md: ICF Int, London School of Hygiene and Tropical Medicine; 2012.

[12] Malm KL, Segbaya S, Forson I, Gakpey KD, Sampong LB, Adjei EA (2013). Implementation of the Affordable Medicines for malaria facility (AMFm) in Ghana: processes, challenges and achievements. J Public Health Policy.

[13] Talisuna AO, Karema C, Ogutu B, Juma E, Logedi J, Nyandigisi A (2012). Mitigating the threat of artemisinin resistance in Africa: improvement of drug-resistance surveillance and response systems. Lancet Infect Dis.

[14] Davis B, Ladner J, Sams K, Tekinturhan E, de Korte D, Saba J (2013). Artemisinin-based combination therapy availability and use in the private sector of five AMFm phase 1 countries. Malar J.

[15] Bryce J, Boschi-Pinto C, Shibuya K, Black RE (2005). WHO child health epidemiology reference group. WHO estimates of the causes of death in children. Lancet.

[16] Larson BA, Amin AA, Noor AM, Zurovac D, Snow RW (2006). The cost of uncomplicated childhood fevers to Kenyan households: implications for reaching international access targets. BMC Public Health.

[17] Matowe L, Adeyi O (2010). The quest for universal access to effective malaria treatment: how can the AMFm contribute. Malar J.

[18] Ansah EK, Narh-Bana S, Affran-Bonful H, Bart-Plange C, Cundill B, Gyapong M (2015). The impact of providing rapid diagnostic malaria tests on fever management in the private retail sector in Ghana: a cluster randomized trial. BMJ.

[19] Adjei GO, Darkwah AK, Goka BQ, Bart-plang C (2009). Parents’ perceptions, attitudes and acceptability of treatment of childhood malaria with artemisinin combination therapies in Ghana. Ghana Med J.

[20] Agyepong IA (1992). Malaria: ethnomedical perceptions and practice in an Adangbe farming community and implications for control. Soc Sci Med.

[21] Sirima SB, Konate A, Tiono AB, Convelbo N, Cousens S, Pagnoni F (2003). Early treatment of childhood fevers with pre-packaged antimalarial drugs in the home reduces severe malaria morbidity in Burkina Faso. Trop Med Int Health.

[22] Chuma J, Okungu V, Molyneux C (2010). Barriers to prompt and effective malaria treatment among the poorest population in Kenya. Malar J.

[23] Laxminarayan R, Over M, Smith DL (2006). Will a global subsidy of new antimalarials delay the emergence of resistance and save lives?. Health Aff (Millwood).

[24] Noor AM, Zurovac D, Hay SI, Ochola SA, Snow RW (2003). Defining equity in physical access to clinical services using geographical information systems as part of malaria planning and monitoring in Kenya. Trop Med Int Health.

[25] Guyatt HL, Snow RW (2004). The management of fevers in Kenyan children and adults in an area of seasonal malaria transmission. Trans R Soc Trop Med Hyg.

[26] Goodman C, Kachur P, Abdulla S, Mwageni E, Nyoni J, Schellenberg JA (2004). Retail supply of malaria-related drugs in rural Tanzania: risks and opportunities. Trop Med Int Health.

[27] Tipke M, Diallo S, Coulibaly B, Störzinger D, Hoppe-Tichy T, Sie A (2008). Substandard anti-malarial drugs in Burkina Faso. Malar J.

[28] WHO. World Malaria Report 2008. Geneva:World Health Organization; 2008. http://www.who.int/malaria/publications/atoz/9789241563697/en/.

